# Effects of short-term heat shock and physiological responses to heat stress in two *Bradysia* adults, *Bradysia odoriphaga* and *Bradysia difformis*

**DOI:** 10.1038/s41598-017-13560-4

**Published:** 2017-10-17

**Authors:** Guodong Zhu, Ming Xue, Yin Luo, Guixia Ji, Fang Liu, Haipeng Zhao, Xia Sun

**Affiliations:** College of Plant Protection, Shandong Agricultural University; Key Laboratory of Biology of Vegetable Pests and Diseases, Shandong Province, 271018 China

## Abstract

*Bradysia odoriphaga* and *Bradysia difformis* are devastating pests of vegetable, ornamental crops and edible mushrooms causing significant losses. Temperature may be an important factor restricting their population abundance in the summer. To determine the effects of short-term heat shock on adults, their survival, longevity and fecundity data were collected, and antioxidant responses and heat shock protein expression levels were examined. Our results indicated that the survival rates of *Bradysia* adults decreased rapidly after heat shock ≥36 °C, and the longevity and reproductive capacities were significantly inhibited, indicating that short-term heat shock had lethal and sub-lethal effects. Moreover, the lipid peroxidation levels of *B. difformis* and *B. odoriphaga* increased dramatically at 36 °C and 38 °C, respectively. Four antioxidant enzymes activities of *B. odoriphaga* were greater than those of *B. difformis* at 38 °C. Additionally, *hsp7*0 and *hsp90* expression levels significantly increased after heat stress, and higher expression levels of *B. difformis* and *B. odoriphaga* were discovered at 36 and 38 °C respectively, indicating their different heat tolerance levels. Overall, short-term heat shock (≥36 °C) caused significantly adverse effects on *Bradysia* adults, indicating that it could be applied in pest control, and antioxidant system and *hsp* genes played important roles in their heat tolerance levels.

## Introduction

Insects, typical small-bodied poikilotherms, are easily affected by environmental factors that limit their abundance and distribution. Temperature is an important abiotic environmental factor that causes the body temperatures of insects to quickly fluctuate to lethal levels, resulting in rapid metabolic variation, which can lead to disorder, affecting survival and fecundity^1,2^. Because of ongoing global warming, the average temperature has increased over the past 30 years, and the frequency and extent of heat events have increased during the summer^3,4^. Thus, interest in the impact of heat stress on insects has grown^5,6^. Heat stress can have rapid lethal effect on insects, which has been widely reported^7,8^. However, understanding the sub-lethal impacts of thermal stress on the development and reproduction of surviving insects could provide important basic information for insect ecology study^9,10^. When the temperature is between the lethal high and physiological limits, insects can be affected by thermal injuries, which result in the loss of life or decline in fecundity^9^. Moreover, because insects can be killed by short exposure to an extremely high temperature, heat treatments can be applied to control horticultural and stored-product pests, with fewer insecticidal applications, decreasing the environmental threat^11,12^. During evolution, insects evolved many behavioral and physiological strategies, such as elevating antioxidant defenses and synthesizing heat shock proteins (Hsps), which are critical indicators in thermal tolerance research, to avoid thermal and other stress impairments^12–15^. An understanding of the impacts of environmental changes on insects and their adaptive mechanisms is vital in studying insect-climate interactions, and will aid in predicting and explaining the regular occurrences of insects in different seasons and regions^16,17^.

In general, there is a balance between reactive oxygen species (ROS) generation and scavenging. However, the balance is disturbed under environmental stress. Thermal stress is responsible for increasing the generation of ROS, which causes oxidative damage^18^. The surplus ROS causes lipid peroxidation (LPO) and disrupts cell membrane fluidity, resulting in cell lesions^18^. The degree of membrane LPO can be determined indirectly by measuring the malondialdehyde (MDA) concentration^19^. To maintain homeostasis and prevent ROS damage, organisms have evolved complex adaptation-related mechanisms for eliminating ROS, including molecular antioxidants and anti-oxidative enzymes^20^. Antioxidative enzymes, including superoxide dismutase (SOD), catalase (CAT), peroxidase (POD) and glutathione-S-transferases (GSTs), are the most important components for protecting cells and maintaining homeostasis involved in various stress conditions by scavenging ROS^21,22^. Many studies have measured antioxidant responses under thermal stress conditions as indicators of the important physiological adaptation processes of insects, including *Corythucha ciliata* ^23^, *Bactrocera dorsalis* ^24^ and *Plutella xylostella* ^25^.

Inducing Hsp (heat shock protein) gene expression levels is an important physiological adaptation to biotic and abiotic stresses. HSPs act as molecular chaperones that participate in maintaining regular cellular functions and in regulating metabolic activity, thereby protecting cells from oxidative damage^14^. Among the different heat shock proteins, Hsp70 and Hsp90 belong to two major conserved families and are commonly expressed under thermal and other stress conditions. In addition to preventing oxidative damage, they may also interfere with the signaling events that trigger the apoptotic process^26^. In previous studies, *hsp90* and *hsp70*, as stress markers, have played important roles in resisting high-temperature stress and in protecting insects from thermal injury and death^14,27^. The different expression levels of *hsp70* and *hsp90* correlate positively with the thermotolerance of insect species and populations^27,28^.

*Bradysia odoriphaga* Yang et Zhang and *Bradysia difformis* Frey, two main root maggot flies, are devastating pests of liliaceous vegetables, flowers and edible fungi, and they can coexist on the same host plant in protected cultivation or in open fields^29–32^. Their larvae tend to aggregate to attack and damage roots and corm tissues, resulting in moisture loss and even death^31,33,34^. In Chinese chive fields, the two *Bradysia* species occur with similar regularities, with outbreaks in the spring, autumn and winter in greenhouses, and population decreases in the summer^35,36^. Temperature was thought to be an important factor affecting their population dynamics during different seasons. The optimum temperature ranges of the two *Bradysia* flies were 13–28 °C for *B. odoriphaga* and 10–25 °C for *B. difformis*, and a temperature over 30 °C had adverse effects on both species. The development threshold temperature (*T*_*0*_) of *B. odoriphaga* was 6.29–8.7 °C, while 4.0–8.4 °C for *B. difformis* ^32,34^, and we hypothesized *B. difformis* had a lower optimum temperature range and threshold temperature, indicating greater cold tolerance than *B. odoriphaga*. Because extreme daytime temperatures can exceed 35 °C for several hours during the summer season in northern China, high temperature was regarded as a critical abiotic factor restricting their occurrence in the summer. However, there are no reports regarding the thermal tolerance levels of the two *Bradysia* flies against heat stress. Our previous work indicated that the two *Bradysia* species were sensitive to heat stress, additionally, adults stage being the most sensitive stage to heat shock (unpublished). Other researchers also confirmed that heat shock negatively influences *B. odoriphaga* ^37^, but no research about heat tolerance of *B. difformis* was reported. To manage root maggot flies efficiently in Chinese chive fields, it is important to clarify the impact of high temperature on the survival and fecundity of these pests, which will aid in predicting their occurrences.

In this study, we demonstrated the lethal and sub-lethal effects of heat shock on two *Bradysia* adults. The physiological responses to heat stress in the two root maggot flies were then determined, including those of the antioxidant systems and *hsp* gene expression levels. Our findings provide an important theoretical basis for predicting population dynamics and understanding the potential physiological adaptations to heat stress for two important *Bradysia* flies.

## Results

### Lethal effects of heat shock

**Figure 1:**
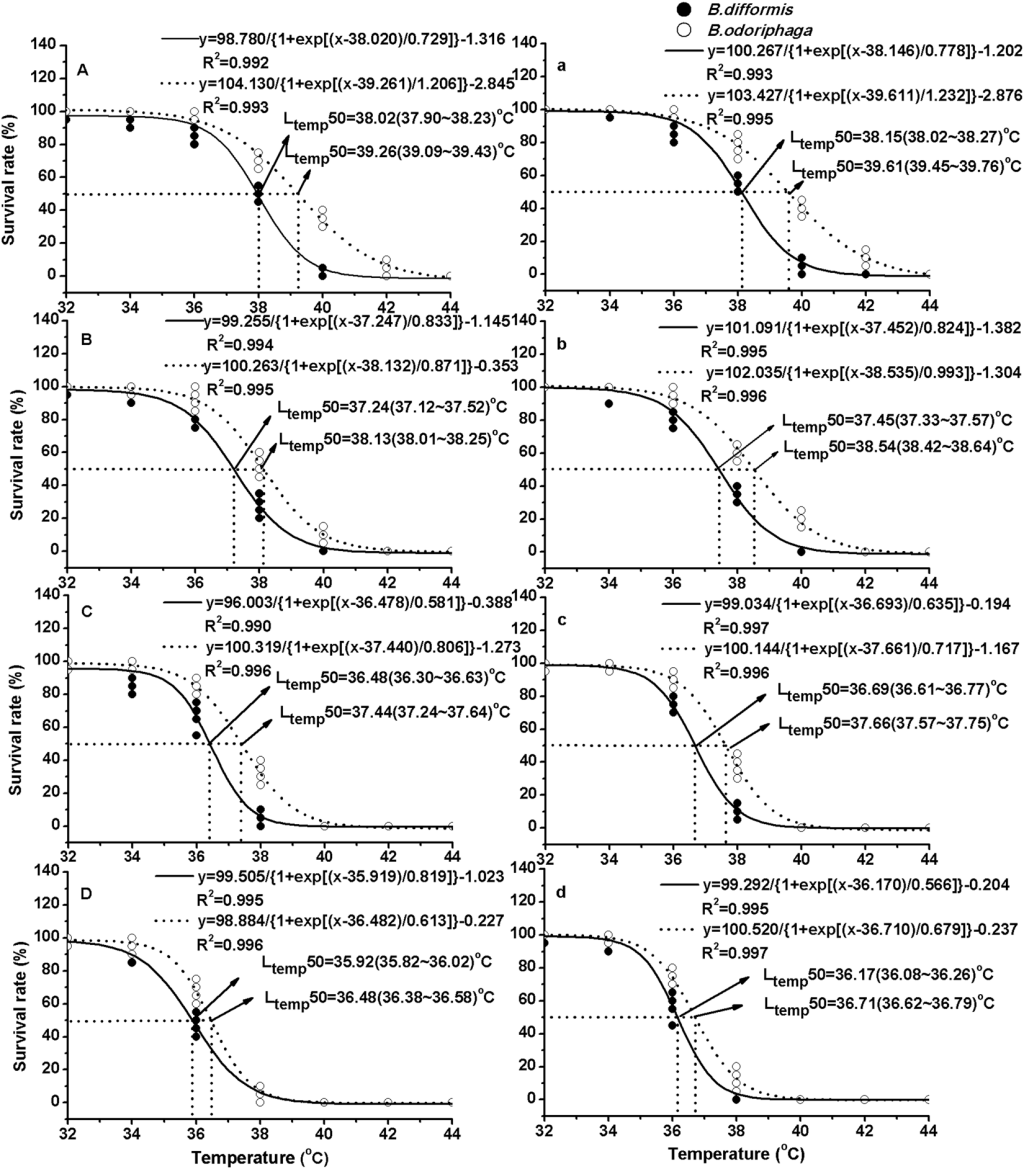
Survival of *B. odoriphaga* and *B. difformis* adults after short-term heat shock. The letters, (**A** and **a**, **B** and **b**, **C** and **c**, **D** and **d**), mean the survival rates after heat shock at 0.5, 1, 2 and 4 h treatment, respectively. (**A**,**B**,**C** and **D**) mean the survival rates of female adults, while (**a**,**b**,**c** and **d**) mean the survival rates male adults. The logistic regression equations indicate the correlation between the treated temperatures (x) and survival rates (y) of two *Bradyisa* adults, and the inflection points of equations indicate the L_temp_50 (the median lethal temperatures) (95%CL) at every treated time.

When the temperature excessed 36 °C, heat shock exerted lethal effects on both *Bradysia* adults (Table 1 and Fig. 1). As an example, the heat shock at 36 °C for 1 h resulted in *B. difformis* survival rates of 80% (female) and 84% (male), while *B. odoriphaga* was not affected. When the temperature increased to 38 °C, the *B. odoriphaga* survival rates were 53% (female) and 62% (male), while those of *B. difformis* were 28% (female) and 34% (male), and at 40 °C, no *B. difformis* survived, while the *B. odoriphaga* survival rates were 11% (female) and 19% (male).

**Table 1:** Survival rate of two *Bradysia* adults after heat shock.

Temperature	Time	Female survival rate (%)					Male survival rate (%)			
		*B. odoriphaga*	*B. difformis*	t	P	*B. odoriphaga*		*B. difformis*	t	P
40 °C	2.0 h	—	—			—		—		
	1.0 h	11.00 ± 1.87 e	—			19.00 ± 1.87 e		—		
38 °C	2.0 h	33.00 ± 2.55 Ad	5.00 ± 1.58 Be	9.333	<0.001	38.00 ± 2.55 Ad		11.00 ± 1.87 Be	8.538	<0.001
	1.0 h	53.00 ± 2.55 Ac	28.00 ± 2.55 Bd	6.934	0.001	62.00 ± 2.00 Ac		34.00 ± 1.87 Bd	10.224	<0.001
36 °C	2.0 h	84.00 ± 1.87 Ab	67.00 ± 3.39 Bc	4.389	0.002	89.00 ± 1.87 Ab		74.00 ± 1.87 Bc	5.669	0.001
	1.0 h	93.00 ± 2.55 Aa	80.00 ± 2.24 Bb	3.833	0.005	95.00 ± 1.58 Aab		84.00 ± 2.92 Bb	3.317	0.011
25 °C	—	100 a	100 a			100 a		100 a		
	df	(5,24)	(4,20)			(5,24)		(4,20)		
	F	290.32	295.25			331.61		358.37		
	P	<0.001	<0.001			<0.001		<0.001		

Each value represents the average (±s.e.) of five replicates. The different capital letters at the first column after each datum indicate significant differences in survival rates of two *Bradysa* adults (same single-sex) (*P* < 0.05) at the same heat stress, and the different small letters at the second column indicate significant differences in survival rates of *B. odoriphaga* or *B. difformis* (*P* < 0.05) among different heat stress conditions. Data was analyzed with Independent-Samples T Test and ANOVA (Tukey’s HSD).

We calculated the L_temp_50 according to the logistic regression (Eq. 1; Fig. 1). As the treatment time was prolonged, the L_temp_50 declined. After a 0.5-h exposure, the L_temp_50 values of *B. odoriphaga* were 39.26 °C (female) and 39.61 °C (male), while those of *B. difformis* were 38.02 °C (female) and 38.15 °C (male), indicating heat shock (≥36 °C) possessing lethal effects. The L_temp_50 values of *B. odoriphaga* were 1.24 and 1.46 °C higher than those of *B. difformis*, which indicated that *B. odoriphaga* was more heat tolerant than *B. difformis*.

### Sub-lethal effects of heat shock

#### Longevity

The mean adult longevity values of the two *Bradysia* species decreased significantly as the exposure time increased at the tested temperature (34, 36 and 38 °C) (Table 2). The female adults longevity of *B. difformis* ranged from 2.27 (34 °C for 0.5 h) to 0.92 (38 °C for 1 h) d, while that of *B. odoriphaga* females ranged from 2.47 (34 °C for 0.5 h) to 0.70 (38 °C for 2 h) d. However, the male adults had greater longevity values than females under the same heat shock condition. No *B. difformis* adults survived 12 h after a heat shock of 38 °C for 2 h, while no *B. odoriphaga* adults survived 12 h after a heat shock of 38 °C for 4 h. After a 36 °C heat shock for 1 h, the longevity values of *B. odoriphaga* adults were 2.16 (female) and 2.71 (male) d, shortened by 0.52 and 0.32 d, respectively, compared with at 25 °C, while the values of *B. difformis* adults were 1.73 (female) and 2.13 (male) d, shortened by 0.95 and 0.83 d, respectively.

**Table 2:** Longevity and reproductive capacity of *B. odoriphaga* and *B. difformis* adults after heat shock.

Temperature	Time	Female adults longevity (d)		Male adults longevity (d)		Fecundity (eggs/ female)		Egg hatching rate (%)		Spawning rate (%)	
		*B. difformis*	*B. odoriphaga*	*B. difformis*	*B. odoriphaga*	*B. difformis*	*B. odoriphaga*	*B. difformis*	*B. odoriphaga*	*B. difformis*	*B. odoriphaga*
38°C	2.0 h	—	0.70 ± 0.06 f	—	1.08 ± 0.08 c	—	29.94 ± 2.89 f	—	34.96 ± 3.83 d	—	50.00 ± 2.89 e
	1.0 h	0.92 ± 0.09 g	0.93 ± 0.07 f	1.31 ± 0.08 f	1.30 ± 0.07 c	25.00 ± 1.13 f	56.49 ± 2.42 e	37.94 ± 4.06 f	46.44 ± 3.71 c	48.33 ± 1.67 d	76.67 ± 1.67 cd
	0.5 h	1.24 ± 0.09 efg	1.40 ± 0.07 e	1.69 ± 0.07 ef	1.79 ± 0.06 b	36.00 ± 1.34 e	84.59 ± 2.47 d	67.21 ± 2.81 d	69.97 ± 3.22 b	70.00 ± 2.89 c	78.33 ± 1.67 cd
36°C	4.0 h	1.07 ± 0.09 fg	1.33 ± 0.10 e	1.51 ± 0.08 f	1.81 ± 0.08 b	24.03 ± 1.04 f	58.43 ± 2.75 e	56.69 ± 2.44 e	68.83 ± 3.14 b	51.67 ± 1.67 d	60.00 ± 2.89 e
	2.0 h	1.47 ± 0.09 def	2.13 ± 0.08 d	1.94 ± 0.08 de	2.73 ± 0.07 a	31.98 ± 1.32 e	97.70 ± 2.15 c	65.89 ± 1.95 d	88.33 ± 1.43 a	70.00 ± 2.89 c	71.67 ± 1.67 d
	1.0 h	1.73 ± 0.10 cd	2.16 ± 0.08 cd	2.13 ± 0.10 cd	2.71 ± 0.07 a	37.39 ± 1.25 de	96.75 ± 2.10 c	80.96 ± 1.36 c	88.45 ± 1.48 a	76.67 ± 1.67 bc	86.67 ± 1.67 bc
	0.5 h	2.09 ± 0.07 bc	2.33 ± 0.06 bcd	2.31 ± 0.09 bcd	2.87 ± 0.07 a	46.84 ± 1.57 cd	108.96 ± 2.03 b	82.66 ± 1.74 c	91.53 ± 1.10 a	86.67 ± 1.67 ab	96.67 ± 1.67 ab
34°C	4.0 h	1.53 ± 0.10 de	2.22 ± 0.07 bcd	2.22 ± 0.11 cd	2.70 ± 0.09 a	42.85 ± 1.40 d	110.82 ± 1.96 b	77.03 ± 2.27 c	87.31 ± 1.45 a	78.33 ± 1.67 bc	93.33 ± 1.67 ab
	2.0 h	1.88 ± 0.11 bcd	2.49 ± 0.06 abc	2.45 ± 0.11 bc	2.98 ± 0.08 a	52.55 ± 1.33 bc	114.53 ± 2.07 ab	85.10 ± 1.72 bc	92.73 ± 1.02 a	85.00 ± 2.89 ab	96.67 ± 1.67 ab
	1.0 h	2.18 ± 0.08 b	2.46 ± 0.06 abc	2.50 ± 0.06 bc	2.98 ± 0.08 a	53.00 ± 1.30 bc	116.25 ± 2.40 ab	92.08 ± 0.80 ab	92.67 ± 0.58 a	96.67 ± 1.67 a	95.00 ± 2.89 ab
	0.5 h	2.27 ± 0.10 ab	2.47 ± 0.06 ab	2.67 ± 0.06 ab	2.96 ± 0.08 a	58.30 ± 1.34 ab	116.54 ± 3.00 ab	92.61 ± 0.78 ab	93.07 ± 1.73 a	93.33 ± 3.33 a	96.67 ± 1.67 ab
25°C	—	2.68 ± 0.06 a	2.68 ± 0.06 a	2.96 ± 0.06a	3.03 ± 0.06 a	62.60 ± 1.25 a	122.83 ± 2.25 a	95.29 ± 0.70 a	95.93 ± 0.64 a	95.00 ± 2.89 a	98.33 ± 1.67 a
	df	(10,649)	(11,708)	(10,649)	(11,708)	(10,499)	(11,588)	(10,514)	(11,708)	(10,22)	(11,24)
	F	36.367	92.548	35.690	88.175	86.879	127.971	77.861	89.185	48.536	62.545
	P	<0.001	<0.001	<0.001	<0.001	<0.001	<0.001	<0.001	<0.001	<0.001	<0.001

Each value represents the mean (±SE) of three replications. Different letters after the data indicate the significant difference (*P* < 0.05) of the same species among different heat stress condition. Data was analyzed with ANOVA (Tukey’s HSD).

#### Reproductive capacity

When the treatment temperature exceeded 34 °C, heat shocks significantly suppressed the reproductive capacities of two *Bradysia* adults (Table 2). The female fecundity of *B. odoriphaga* ranged from 108.96 (36 °C for 0.5 h) to 29.94 eggs (38 °C for 2 h), while that of *B. difformis* ranged from 46.84 (36 °C for 0.5 h) to 25.00 eggs (38 °C for 1.0 h). The 1-h treatment at 36 °C resulted in *B. odoriphaga* fecundity of 96.75 eggs, declining by 21.23% compared with at 25 °C, while that of *B. difformis* was 25.00 eggs, declining by 40.27%. Heat shock at 34 °C exerted slight effects.

Additionally, after heat shock partial female adults could not lay eggs (Table 2). After 1-h exposures at 36 °C and 38 °C, the spawning rates of *B. odoriphaga* were 86.67% and 76.67%, respectively, while those of *B. difformis* were 76.67% and 48.33%, respectively.

Although eggs were oviposited successfully, the hatching rates were significantly inhibited (Table 2). At 36 °C with exposure lengths from 0.5 to 4 h, the hatching rate of *B. odoriphaga* ranged from 91.53% to 68.83%, while that of *B. difformis* ranged from 82.66% to 56.69%.

### Antioxidant responses

#### Lipid peroxidation (LPO)

**Figure 2:**
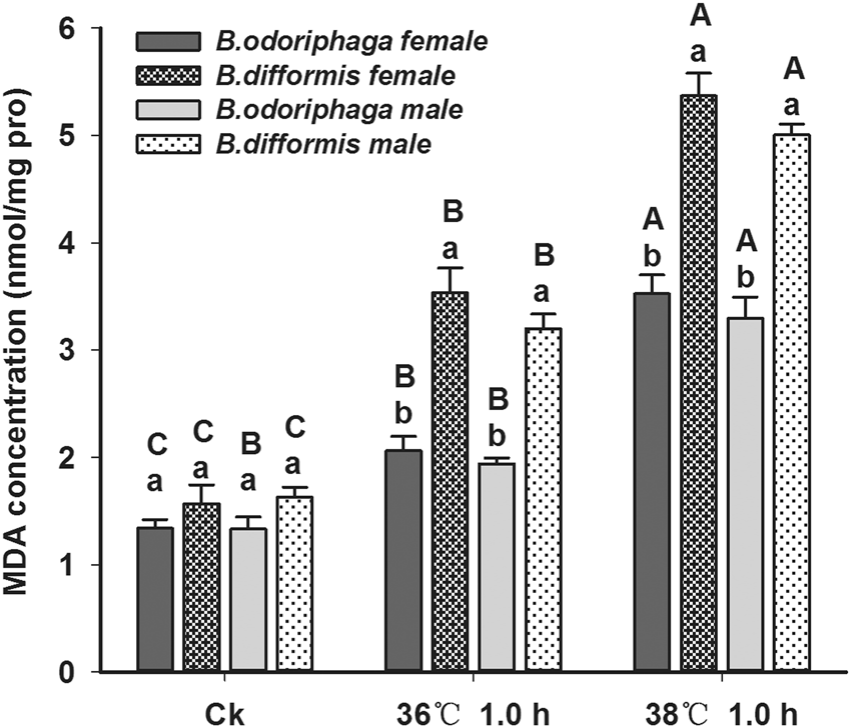
Malondialdehyde (MDA) concentrations in *B. odoriphaga* and *B. difformis* adults after heat shock. Each value represents the average (±s.e.) of three replicates. Different capital letters over the bars indicate significant differences in MDA concentrations of *B. odoriphaga* or *B. difformis* (same single-sex) among different heat stress conditions (*P* < 0.05), while and the different small letters indicate significant differences of two *Bradysia* adults at the same heat stress (*P* < 0.05). Data was analyzed with ANOVA (Tukey’s HSD).

The MDA concentrations in *B. odoriphaga* (female, F_2,6_ = 61.818, P < 0.001; male, F_2,6_ = 48.793, P < 0.001) and *B. difformis* (female, F_2,6_ = 85.546, P < 0.001; male, F_2,6_ = 243.311, P < 0.001) increased significantly after heat stress (Fig. 2). The MDA concentration in *B. difformis* began to increase significantly at 36 °C, while that of *B. odoriphaga* began to increase significantly at 38 °C. Additionally, at 36 °C and 38 °C, the MDA concentration in *B. difformis* was greater than in *B. odoriphaga* adults (F_3,8_ = 27.352, P < 0.001 at 36 °C; F_3,8_ = 34.797, P < 0.001 at 38 °C).

#### Antioxidant enzyme activities

CAT activities of *B. odoriphaga* (female, F_2,6_ = 11.090, P = 0.010; male, F_2,6_ = 33.927, P = 0.001) and *B. difformis* (female, F_2,6_ = 15.524, P = 0.004; male, F_2,6_ = 27.598, P = 0.001) adults exhibited significant changes in response to heat stress (. 3 A). Compared with at 25 °C, the CAT activities of *B. odoriphaga* increased by 64% (female) and 90% (male) at 36 °C for 1 h, and those of *B. difformis* increased by 55% (female) and 73% (male) indicating the higher CAT activities of *B. odoriphaga* than *B. difformis* (F_3,8_ = 10.511, P = 0.004). At a greater heat stress (38 °C), the CAT activities of *B. difformis* declined compared with at 25 °C, while those of *B. odoriphaga* declined compared with at 36 °C but still higher than at 25 °C.

**Figure 3:**
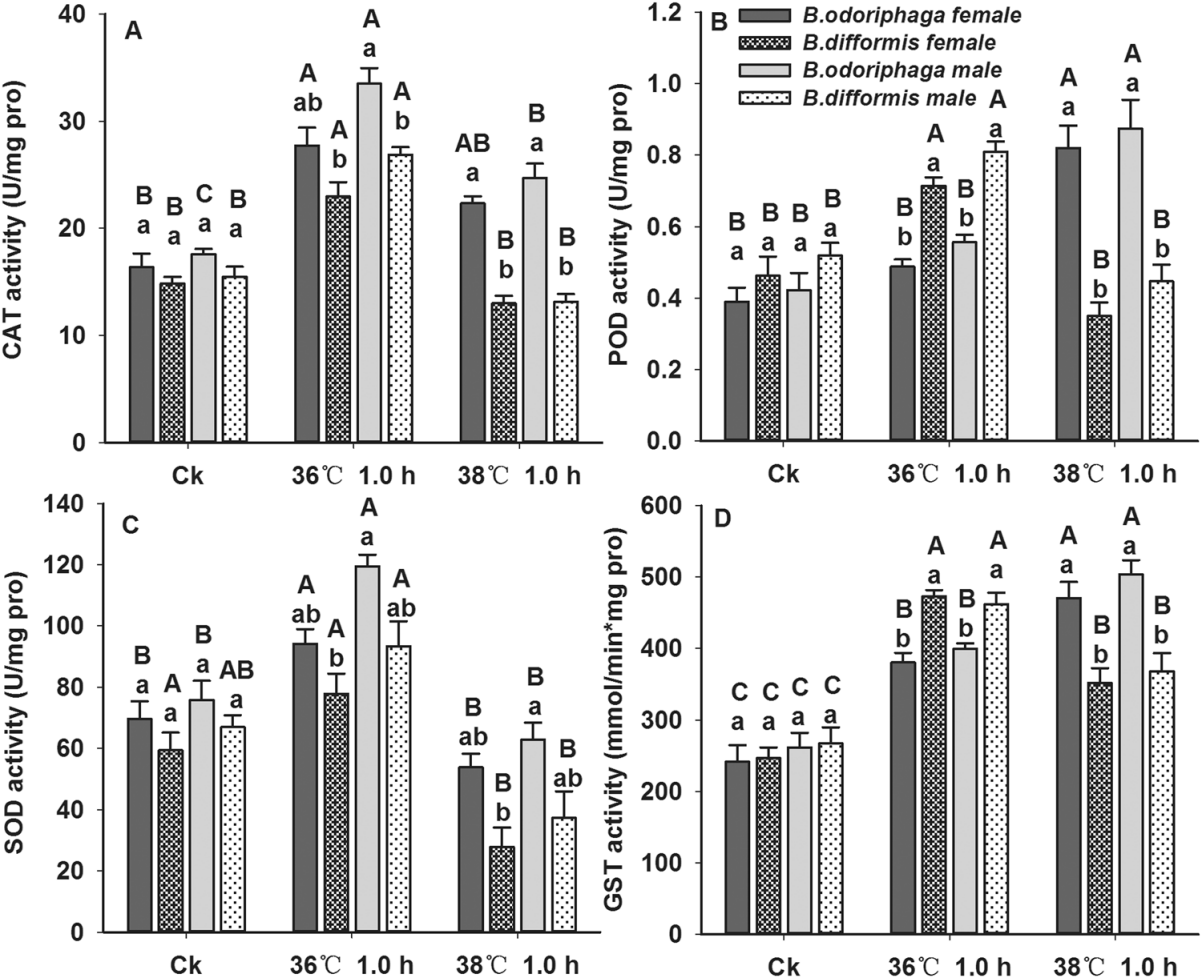
CAT (**A**), POD (**B**), SOD (**C**), GST (**D**) activities of *B. odoriphaga* and *B. difformis* adults after heat shock. Each value represents the average (±s.e.) of three replicates. Different capital letters over the bars indicate significant differences in enzyme activities of *B. odoriphaga* or *B. difformis* (same single-sex) among different heat stress conditions (*P* < 0.05), while and the different small letters indicate significant differences of two *Bradysia* adults at the same heat stress (*P* < 0.05). Data was analyzed with ANOVA (Tukey’s HSD).

POD activities of *B. odoriphaga* (female, F_2,6_ = 14.352, P = 0.005; male, F_2,6_ = 10.744, P = 0.010) and *B. difformis* (female, F_2,6_ = 12.022, P = 0.008; male, F_2,6_ = 12.826, P = 0.007) adults were affected by heat shock (Fig. 3B). The maximum POD activity levels in *B. difformis* and *B. odoriphaga* occurred at 36 °C and 38 °C, respectively. At 36 °C, the POD activities of *B. difformis* were higher than those of *B. odoriphaga* (F_3,8_ = 38.317, P < 0.001), unlike at 38 °C (F_3,8_ = 19.731, P < 0.001). Noticeably, compared with at 25 °C, POD activities of *B. difformis* were inhibited at 38 °C.

SOD activities were significantly influenced by heat stress (*B. odoriphaga* female, F_2,6_ = 16.711, P = 0.004, male, F_2,6_ = 31.770, P = 0.001; *B. difformis* female, F_2,6_ = 16.364, P = 0.004, male, F_2,6_ = 14.974, P = 0.005) (Fig. 3C). At 36 °C, the SOD activities of *B. odoriphaga* increased by 35% (female) and 57% (male) compared with at 25 °C, while those of *B. difformis* increased by 31% (female) and 40% (male). At 38 °C, the SOD activities of the two species were significantly inhibited compared with at 25 °C, while the activities of *B. odoriphaga* were still greater than those of *B. difformis*.

After exposure to 36 and 38 °C, the GST activities of the two species were significantly enhanced (*B. odoriphaga* female, F_2,6_ = 16.711, P = 0.004, male, F_2,6_ = 31.770, P = 0.001; *B. difformis* female, F_2,6_ = 16.364, P = 0.004, male, F_2,6_ = 14.974, P = 0.005) (Fig. 3D). The highest GST activities in *B. difformis* and *B. odoriphaga* occurred at 36 °C and 38 °C, respectively. Compared with at 25 °C, the GST activities of *B. difformis* increased by 75% (female) and 80% (male) at 36 °C, which were greater than those of *B. odoriphaga* (F_3,8_ = 14.954, P = 0.001). Conversely, the GST activities of *B. odoriphaga* were higher than those of *B. difformis* at 38 °C.

### Hsp gene expression

#### hsp70

**Figure 4:**
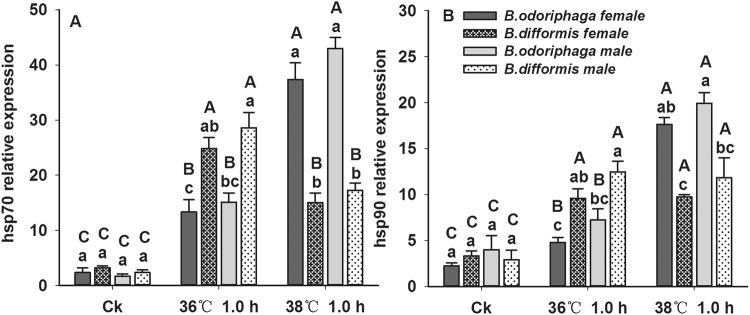
Changes in *hsp70* (**A**) and *hsp90* (**B**) expression of *B. odoriphaga* and *B. difformis* adults after heat shock. Each value represents the average (±s.e.) of three replicates. Different capital letters over the bars indicate significant differences in gene expression levels of *B. odoriphaga* or *B. difformis* (same single-sex) among different heat stress conditions (*P* < 0.05), while and the different small letters indicate significant differences of two *Bradysia* adults at the same heat stress (*P* < 0.05). Data was analyzed with ANOVA (Tukey’s HSD).

The *hsp70* expression levels in *B. odoriphaga* (female, F_2,6_ = 65.280, P < 0.001; male, F_2,6_ = 186.099, P < 0.001) and *B. difformis* (female, F_2,6_ = 49.447, P < 0.001; male, F_2,6_ = 52.535, P < 0.001) adults were significantly induced by heat stress (Fig. 4A). At 36 °C, the *hsp70* expression in *B. odoriphaga* was up-regulated by 4.66- (female) and 7.84- (male) fold, while in *B. difformis* it was up-regulated by 6.72- (female) and 11.08- (male) fold, compared with at 25 °C. In addition, the maximal inductions in *B. difformis* and *B. odoriphaga* were obtained at 36 °C and 38 °C, respectively.

#### hsp90

*hsp90* expression levels in *B. odoriphaga* (female, F_2,6_ = 217.970, P < 0.001; male, F_2,6_ = 40.782, P < 0.001) and *B. difformis* (female, F_2,6_ = 15.524, P = 0.004; male, F_2,6_ = 12.012, P = 0.008) adults increased dramatically at the treatment temperatures (Fig. 4B). Compared with at 25 °C, *hsp90* expression in *B. odoriphaga* was up-regulated by 1.13- (female) and 0.81- (male) fold at 36 °C, while in *B. difformis* it was up-regulated by 1.87- (female) and 3.25- (male) fold. Consistent with the *hsp70* results, the maximal inductions of *hsp90* in *B. difformis* and *B. odoriphaga* were recorded at 36 and 38 °C, respectively.

## Discussion

Poikilotherms are usually exposed to various challenges to survival and reproduction in their environments, and temperature is a critical abiotic factor that causes physiological changes in arthropods^24^. When the temperature exceeds the optimum for insects, short-term heat shock can cause rapid death. In this study, heat shock temperatures over 36 °C exerted significant lethal effects. For all treatments (0.5–4 h), the L_temp_50 of *B. odoriphaga* was in the range of 36.48–39.61 °C, and that of *B. difformis* was 35.92–38.15 °C. In northern China, the temperature is normally above 35 °C during the summer months, and the maximum daytime temperature even exceeds 40 °C (Climate Databases, Chinese Academy of Forestry)^37^. Thus, this environmental condition is extremely adverse to *Bradysia* species. Because it absorbs the solar radiation, the ground’s surface temperature is greater than the atmospheric temperature, which could aggravate the heat stress. Previous studies confirmed that *Bradysia* adults, having weak flight capabilities, were mainly active on the ground^32,38^. Thus, the two *Bradysia* adults were bound to confront thermal stress in the summer, and the thermal stress resulted in rapid death, restricting their abundance.

In addition to rapid lethal effects, heat stress also exerted various biological stresses on surviving insects, such as suppressing fecundity and longevity^1,10^. Previous studies confirmed that two *Bradysia* adults did not oviposit at once after emergence, and the pre-oviposition period ranged from 1.0 to 1.5 d^32–34^, indicating that they were restricted to suffer the heat stress in the daytime before oviposition. Results obtained in this study revealed that heat shocks (36–38 °C for 0–4 h) suppressed the reproductive capacity and longevity of the surviving adults. After exposure to 38 °C for 2 h, few *B. difformis* survived, and those that did were unable to oviposit and mate. Meanwhile, more *B. odoriphaga* adults survived, but their fecundity was low. Our findings were partially consistent with the previous reports about other insects. Cheng *et al*. also found heat shock, except for rapid lethal effects, also resulted in longevity and fecundity suppression of *B. odoriphaga* adults^37^. *Agasicles hygrophila* adults suffered adverse effects after being exposed to 36 and 39 °C for 4.0 h, with the fecundity and offspring hatching rate decreasing^39^. Similarly, the longevity of *Helicovrpa armigera* was shortened under heat shock (40–46.5 °C) and the fecundity decreased^40^. The effects of heat stress on fecundity and longevity in insects may be a result of direct injuries to reproductive systems or metabolic disorders. However, we could not determine whether the reduction in fecundity resulted from damage to the reproductive systems of both sexes or to only one sex.

Heat stress (36–40 °C) exerted significant lethal and sub-lethal effects on two *Bradysia* adults, but there were significant differences in the heat-tolerance responses between them: *B. odoriphaga* possessed more heat tolerance than *B. difformis* indicating that the former maintained a higher survival rate after heat shock exposure and suffered less severe sub-lethal effects. Previous research confirmed that *Bemisia tabaci*, whose population peaked in summer, did not exhibit significant negative changes after a 1-h heat shock (37–45 °C), while the fecundity of *Trialeurodes vaporariorum*, whose population peaked under cooler conditions, decreased rapidly^10^. Thermal adaptability limits the distribution and abundance of *Culicoides imicola* and *Culicoides bolitinos*. Compared with *C. imicola*, *C. bolitinos* has a wider altitude range and has stronger heat- and cold-stress tolerance levels^41^. Moreover, *B. dorsalis*, a widely distributed species, whose heat tolerance is enhanced by heat hardening at 35 °C, 37 °C, 39 °C and 41 °C, has a greater thermal plasticity than *Bactrocera correcta*, a narrowly distributed species, whose heat tolerance is only enhanced at 39 °C and 41 °C^42^. Here, the regional distributions of the two *Bradysia* species in Chinese chive fields were significantly different. *B. odoriphaga* has a wide distribution in North China, especially in Shandong, Hebei and Beijing, while *B. difformis* is mainly distributed in the northwest and northeast of China^31,32,36^. We hypothesized that the different responses to heat shock were related to the population dynamics of the two *Bradysia* species.

Generally, exposure to high- or low- temperature stress may lead to oxidative damage and generate surplus ROS in insects^43,44^. To relieve the adverse oxidative stress, insects increase antioxidant defense to maintain a balance in ROS metabolism^45^. For example, the antioxidant enzyme activities (SOD, POD, CAT and GSTs) of *Bactrocera dorsalis* ^24^, *Chilo suppressalis* ^44^, *Antheraea mylitta* ^46^ and *Propylaea japonica* ^47^ were induced by heat stress to protect the insects. In this study, after 36 and 38 °C heat shock treatments, the MDA concentration of *B. difformis* was greater than that of *B. odoriphaga*, which indicated that *B. difformis* suffered more oxidative stress. Moreover, the antioxidant enzyme activities (SOD, POD, CAT and GSTs) varied significantly after heat stress, indicating the protection function of antioxidant enzymes. At 36 °C, the POD, CAT and GST activities of *B. difformis* were greater than those of *B. odoriphaga*, while all of the tested antioxidant enzyme activities of *B. odoriphaga* were greater than those of *B. difformis* at 38 °C. This phenomenon was consistent with that *B. odoriphaga* possessed a stronger heat tolerance than *B. difformis*. Furthermore, the POD and GST activities of *B. odoriphaga* were induced at a higher temperature (38 °C), suggesting that they were stimulated to protect insects by scavenging ROS at a higher heat stress. Meanwhile, the reduction in the SOD activities in both *Bradysia* adults at 38 °C, compared with the control, suggested that excessive ROS could decrease SOD activity. Thus, the different heat tolerance levels in the two *Bradysia* species were related to the different responses of antioxidant enzymes to heat stress.

Hsps of insects are involved in physiological responses to various environmental stresses, especially heat and cold stress^15,42^. Previous research confirmed that Hsp70 and Hsp90 were two prominent Hsps that play important roles in thermal stress. Our study also confirmed that in both *Bradysia* adults the expression levels of *hsp70* and *hsp90* were induced by heat stress, suggesting that these two Hsps were involved in protecting *Bradysia* adults from thermal stress. At 36 °C, the relative expression of *hsp70* and *hsp90* in *B. difformis* increased more significantly than in *B. odoriphaga*, while the opposite was true at 38 °C. Previous studies indicated that the temperature for the onset of the induction of *hsp* gene expression (*T*_*on*_) and the temperature for the maximal induction of gene expression (*T*_*max*_) of *hsp* may be useful indicators to evaluate the thermal tolerance of insects^48,49^. The higher of T_on_ and T_max_ of *hsps* are, the stronger heat tolerance of insects is, while the lower of Ton and Tmax of hsps are, the stronger cold tolerance of insects is. The *T*_*on*_ and *T*_*max*_ of five *hsps* (*hsp20*, *hsp40*, *hsp60*, *hsp70* and *hsp90*) in *Liriomyza huidobrensis*, which possesses a great heat tolerance, were higher than in *Liriomyza sativae*, which has a greater cold tolerance^49^. Similarly, *Drosophila virilis*, the low-latitude species, possesses a greater heat tolerance than *Drosophila lummei*, the high-latitude species, and the *T*_*max*_ of the expression levels of the *hsp* genes in the former were greater than in the latter^28^. Thus, in the current study, we hypothesized that the T_on_ and T_max_ of *hsp70* and *hsp90* in *B. odoriphaga* were close to 38 °C, higher than those in *B. difformis*, close to 36 °C. Indeed, *B. odoriphaga* possessed a greater heat-tolerance than *B. difformis*. Moreover, the synthesis of Hsps and antioxidant enzyme proteins consume biological energy^50^. Thus, the declines in the fecundity and longevity of the *Bradysia* species may have resulted from a reduction in energy, which was consumed to synthesize stress proteins or supporting enzymatic reactions.

In conclusion, our results confirmed that two *Bradysia* adults were sensitive to heat shock and that after short-term heat shocks their longevity and fecundity were suppressed. *B. odoriphaga* possessed the greater heat tolerance, and the difference in the heat tolerance levels between species was related to protective physiological responses, such as antioxidant capacities and *hsp* expression levels.

## Methods

### Insect materials

*Bradysia odoriphaga* Yang and Zhang and *Bradysia difformis* Frey colonies were originally obtained from a Chinese chive greenhouse field in Tai’an, Shangdong, China, in April 2015. Insect colonies were maintained in the Shandong Provincial Key Laboratory of Applied Microbiology, and reared on Chinese chives for more than 5 generations according to the breeding method^32,34^. Eggs, larvae and pupae were reared in culture dishes (Φ = 9 cm) covered with wet filter paper, and newly emerged adults were placed in pairs in oviposition containers (3-cm diameter × 1.5-cm height). Insect colonies were maintained in growth cabinets at 25 ± 1 °C with 75 ± 5% relative humidity, and a 12:12 h light:dark cycle.

### Heat shock treatment

The treatment methods refer to the methods described by Huang *et al*.^9^. Adults (single-sex) that emerged from pupae within a 12-h period were collected in a 10-mL centrifuge tube, and exposed to a water bath at the target temperature (32, 34, 36, 38, 40, 42 and 44 °C) for 0.5, 1, 2 and 4 h. They were allowed to recover at 25 °C for 1 h. The survival number was recorded. The treatment kept at 25 °C was regarded as the control. Each treatment contained 100 individuals for five replicates, and each replicate contained 20 individuals. The median lethal temperatures, L_temp_50 values, were calculated according to the logistic regression (1).

1 $$y\,=\frac{{\rm{A}}1-{\rm{A}}2}{\{1+\exp [\frac{x\,-\,x0}{{\rm{d}}x}]\}}+A2$$

### Longevity and reproductive capacity

Above lethal experiments indicated that 34 °C was the highest temperature exerted no lethal effects on adults within 4 h, while almost no *B. difformis* adults survived at 40 °C. Therefore, after the heat shock (34, 36 and 38 °C for 0.5, 1, 2 and 4 h), the surviving adults were collected as the tested insects. Males and females were paired and placed on oviposition plastic and reared at 25 °C. Adults were checked every 12 h, and the numbers of eggs were recorded until all of the adults died. The average longevity, fecundity and female fertility rate were calculated. The treatment kept at 25 °C was regarded as the control. Every treatment contained 60 pairs of adults for three replicates, and each replicate contained 20 pairs.

### Antioxidant responses and *hsp70* and *hsp90* expression levels

#### Heat treatment

Above lethal experiments indicated that there were significant differences in survival rate of two *Bradysia* adults at 36 and 38 °C for 1 h. The new adults (single-sex) were exposed to a water bath at the target temperature (36 and 38 °C) for 1 h, and then allowed to recover at 25 °C for 1 h. The treatment kept at 25 °C was regarded as the control. All of the surviving adults were flash frozen in liquid nitrogen and stored at −80 °C.

#### Sample preparation and enzyme activity assay

The treated adults were homogenized in a cold mortar with a pestle in 0.05 M phosphate buffer solution, pH 7.8, containing 0.1 mM ethylenediamine tetraacetic acid and 1% polyvinylpyrrolidone. The crude homogenates were centrifuged at 10,000 g for 15 min at 4 °C. The supernatant was gathered for the determination of antioxidant enzyme activities. Protein concentrations were determined using the Bradford assay. The activities of CAT, POD, SOD and GSTs, and the MDA concentration, were determined by spectrophotometry. All spectrophotometric analyses were conducted in a Shimadzu UV-2450 spectrophotometer (Shimadzu, Arlington, MA, USA). Every treatment contained 240 individuals (single-sex) for three replicates, and each replicate contained 80 individuals.

CAT activity was calculated by measuring the consumption of H_2_O_2_ at 240 nm for 2 min. The amount (μmol) of H_2_O_2_ decomposition per min per mg protein was defined as one unit of CAT activity. The result was expressed as U mg^−1^ protein.

POD activity was assayed using the guaiacol oxidation method at 470 nm. One unit of POD activity was defined as the amount that catalyzes 1 μmol substrate reaction per minute per mg protein. The result was expressed as U mg^−1^ protein.

SOD activity was measured based on the inhibition of the nitro blue tetrazolium photochemical reaction at 550 nm. One unit of SOD activity was defined as the amount of enzyme that caused a 50% inhibition of the nitro blue tetrazolium reduction. The result was expressed as U mg^−1^ protein.

GSTs activity was determined using 1-chloro-2,4-dinitrobenzene and reduced glutathione as the substrate. The change in absorbance was measured continuously for 4 min at 340 nm. Changes in absorbance per min were converted into mmol 1-chloro-2,4-dinitrobenzene conjugated/min/mg protein using a molar extinction coefficient of 9.6 mM^−1^ cm^−1^. The result was expressed as mmol min^−1^ mg^−1^ protein.

The LPO was determined indirectly by measuring the amount of MDA formed by reacting with thiobarbituric acid to give a red species having a maximum absorption at 532 nm at 37 °C^25^. The MDA concentration was expressed as nmol of MDA produced per mg protein.

#### Reverse transcriptase-quantitative polymerase chain reaction (RT-qPCR)

Total RNAs were extracted using an RNApure Tissue Kit (DNase I) (ComWin Biotech, Beijing, China). cDNA was synthesized using the SYBR1 PrimeScript RT-qPCR Kit II (Takara Biotechnology, Dalian, China). *hsp70* and *hsp90* mRNA levels were measured using RT-qPCR. RT-qPCR reactions were performed using the Bio-rad CFX96 Real-Time PCR System (BioRad Laboratories, Hercules City, CA, USA) with SYBR-Green detection. The average threshold cycle (Ct) was calculated per sample. The relative expression levels were calculated with the 2^−ΔΔCT^ method. The relative level of each *hsp* was defined as the increase (in folds) compared with the amount of β-actin. RT-qPCR primers and the list of accession numbers are provided (Table 3). The process of how to design these primers was supplied in the Supplementary section (Table 1S, Figs 1S and 2S). Each gene was analyzed in triplicate in each of three biologically independent treatments. Every treatment contained 150 individuals (single-sex) for three replicates, and each replicate contained 50 individuals.

**Table 3:** Primers used in reverse transcriptase-quantitative polymerase chain reaction (RT-qPCR).

Gene	Species	Primer sequence (5′ → 3′)	Fragment length (bp)	GeneBank accession number
hsp70	B. odoriphaga	GACAAACGGCAGATCGAC	114	MF567364
		ATCGGGATTGATCGATAAGTT		
	B. difformis	TCGAGTGGCTATGAATCC	120	MF567365
		ACCACTGTGAATGGCCAA		
hsp90	B. odoriphaga	CATCCCAGTACGGTTGGTC	96	MF567366
		TTTGCCAGCCATGTAACC		
	B. difformis	GAAGGCCAGAAACACATT	113	MF567367
		CGACATACTCATCAATTGG		
β-actin	B. odoriphaga	GAGATGACACAAATCATG	120	MF567368
		AGATTGGTACGGTGTGAG		
	B. difformis	ATGTTTGAACCTTCAACT	134	MF567369
		GACCAGCCAAGTCCAAAC		

### Data analysis

In the logistic regression analysis (Eq. 1), the survival rates of these two *Bradyisa* adults after heat shock were regarded as the dependent variable (y), while the treated temperatures were regarded as the independent variables, and ×0 indicates the L_temp_50 value. We tested the variables for homogeneity of group variances using Levene’s test and normality using the Kolmogorov-Smirnov test prior to statistical analysis. For analysing the difference among different treatments, the survival rate, longevity, fecundity, egg hatching rate and female spawning rate of *B. odoriphaga* or *B. difformis* at each heat treatment were regarded as the dependent variable, while the treatment were regarded as the independent variables in one-way ANOVA followed by Tukey’s HSD multiple comparisons. For analysing the difference between two species, the survival rate, longevity (single-sex), fecundity, egg hatching rate and female spawning rate of *B. odoriphaga* or *B. difformis* at same heat condition were regarded as the Test variables, while the species were regarded as the Group variables in Independent-Samples T Test comparison.

Similarly, when analysing the enzyme activities and gene expression levels of two *Bradysia* adults, the MDA concentration, enzyme activities (CAT, SOD, POD and GSTs) and gene expression levels (*hsp70* and *hsp90*) of *B. odoriphaga* or *B. difformis* at each heat treatment were regarded as the dependent variable, while the treatments were regarded as the independent variables in one-way ANOVA followed by Tukey’s HSD multiple comparisons. With regards to the difference analysis at the same heat conditions, the values of two *Bradyisa* species were regarded as the dependent variable, while the species (single-sex) were regarded as independent variables in the above-mentioned method. All analyses were performed with PASW Statistics 18.0.0 (2009; SPSS Inc. Quarry Bay, HK). Figures were constructed using SigmaPlot 12.0.

### Data availability

All data generated or analysed during this study are included in this published article.

## Electronic supplementary material

Supplementary
