# Supplementary material for: Effects of short-term heat shock and physiological responses to heat stress in two Bradysia adults, Bradysia odoriphaga and Bradysia difformis
Source: Sci Rep. 2017 Oct 17;7:13381. doi: 10.1038/s41598-017-13560-4 (PMC5645341; doi:10.1038/s41598-017-13560-4)
Supplement: Supplementary file 1 — Supplementary [file 41598_2017_13560_MOESM1_ESM.pdf]

Effects of short-term heat shock and physiological responses to heat stress in two *Bradysia* adults, *Bradysia odoriphaga* and *Bradysia difformis*

Guodong Zhu[1], Ming Xue[1*], Yin Luo[1], Guixia Ji[1], Fang Liu[1], Haipeng Zhao[1], Xia Sun[1]

[1] College of Plant Protection, Shandong Agricultural University; Key Laboratory of Biology of Vegetable Pests and Diseases, Shandong Province, 271018, China.

* Corresponding author

E-mail: xueming@sdau.edu.cn

Supplementary:

Methods

Cloning the cDNA section of *hsp70* and *hsp90*

Total RNAs were extracted using an RNApure Tissue Kit (DNase I) (ComWin Biotech, Beijing, China). cDNA was synthesized using the SYBR1 PrimeScript RT-qPCR Kit II (Takara Biotechnology, Dalian, China). The degenerate primers of *hsp70, hsp90* and *β-actin*(Table S1), which were used to amplify the partial segments, were designed according to the conserved amino acid sequences of other insects, such as *Aedes aegypti*, *Anopheles darling*, *Drosophila buzzatii*, *Drosophila melanogaster*, *Stratiomys singularior*, *Exangerona prattiaria*, *Liriomyza sativae*, *Spodoptera exigua*.

Table S1 The degenerate primers of *hsp70, hsp90* and *β-actin* in the cDNA cloning

Gene	Primer sequence (5′→3′)	Fragment length (bp)
hsp70	AGATYATYGCCAAYGACCAG<br>CGDCCCTTGTCGTTCTTGAT	1450
hsp90	AAGCACTCBCARTTCATY<br>RTGGTCAGGRTTRATTTC	1278
β-actin	CAGWSCAAGMGWGGTATCCT<br>GGATCTTCATSAGGTAGTCRG	367

The partial segments of *hsp70* and *hsp90* were sequenced by BioSune Co., Ltd. (Shanghai, China) with Appled Biosystems ABI 3730XL (USA). The sequencing results of gene were submitted to Genebank. The fluorescence primers were designed according to the DNA. The standard curves (Fig. 1S) of the fluorescence primers of *hsp70, hsp90 and β-actin* of two Bradysia species were supplied.

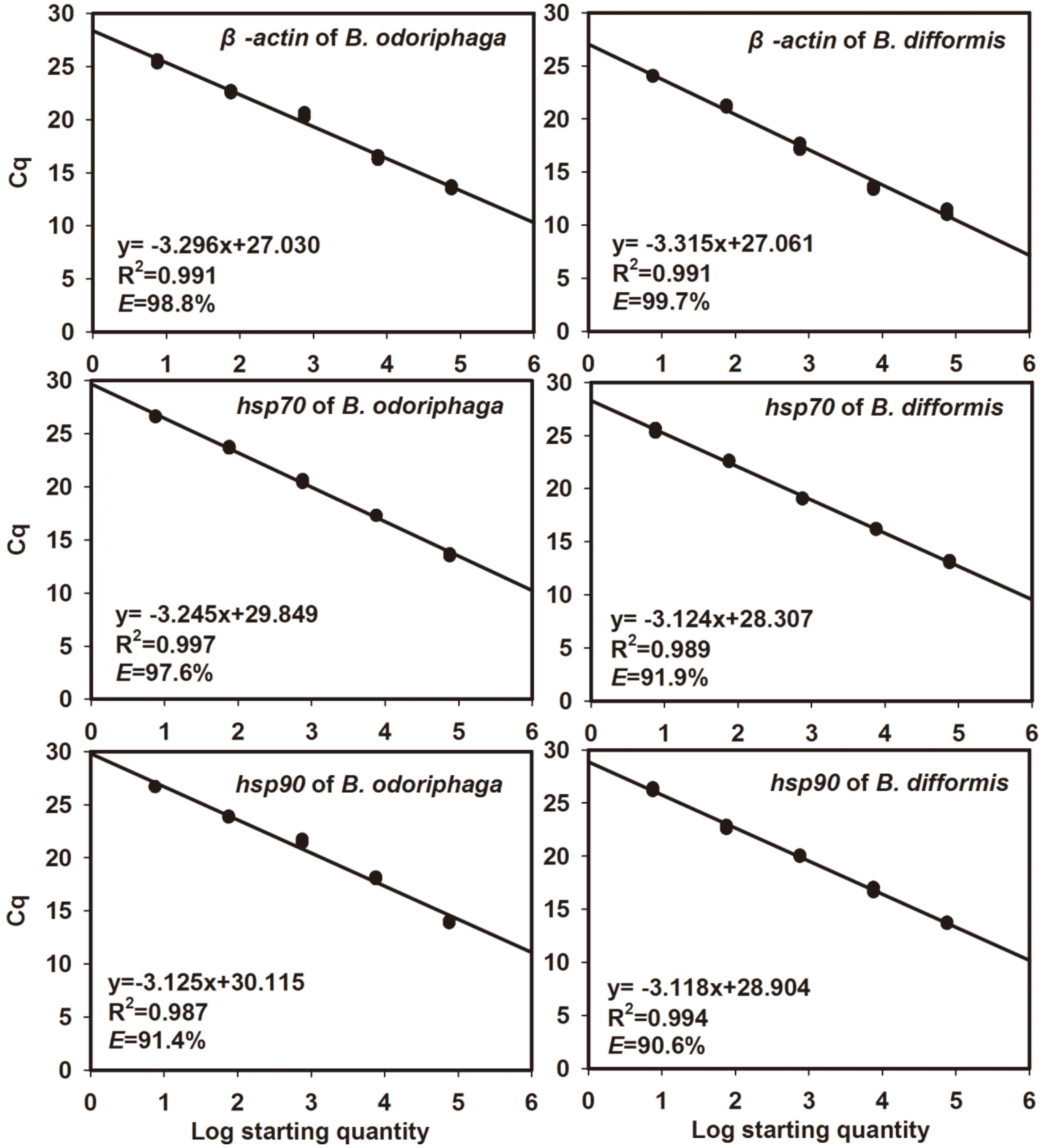

Figure 1S The standard curves of the fluorescence primers of *hsp70, hsp90 and β-actin* of two Bradysia species

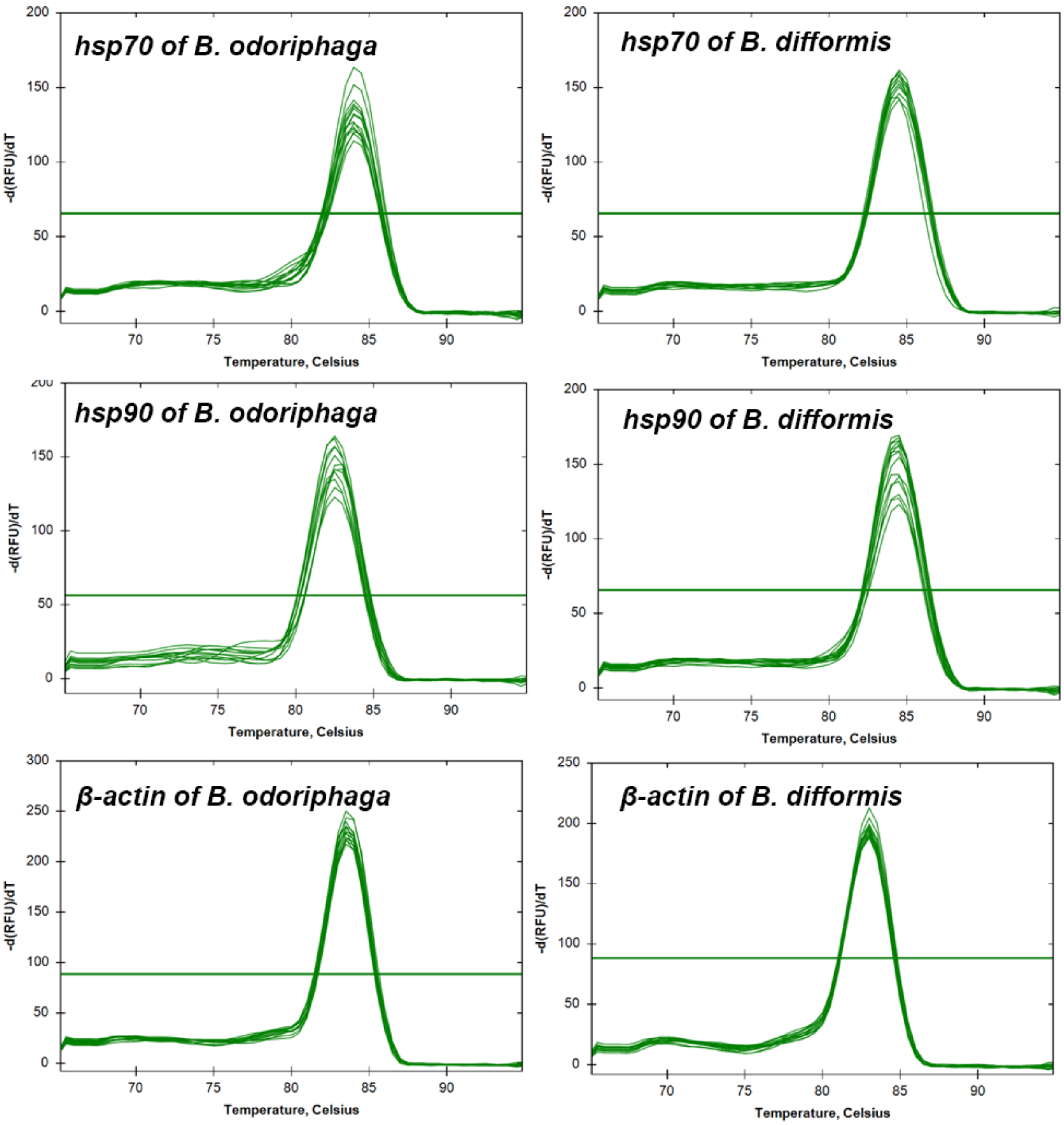

Figure 2S The Melt Curve of fluorescence primers for *hsps* and *β-actin* of two Bradysia Species
